# Reading the Mind in the Eyes or Reading between the Lines? Theory of Mind Predicts Collective Intelligence Equally Well Online and Face-To-Face

**DOI:** 10.1371/journal.pone.0115212

**Published:** 2014-12-16

**Authors:** David Engel, Anita Williams Woolley, Lisa X. Jing, Christopher F. Chabris, Thomas W. Malone

**Affiliations:** 1 Massachusetts Institute of Technology, Sloan School of Management, Cambridge, Massachusetts, United States of America; 2 Carnegie Mellon University, Tepper School of Business, Pittsburgh, Pennsylvania, United States of America; 3 Union College, Department of Psychology, Schenectady, New York, United States of America; 4 Massachusetts Institute of Technology, Center for Collective Intelligence, Cambridge, Massachusetts, United States of America; University of Tuebingen Medical School, Germany

## Abstract

Recent research with face-to-face groups found that a measure of general group effectiveness (called “collective intelligence”) predicted a group’s performance on a wide range of different tasks. The same research also found that collective intelligence was correlated with the individual group members’ ability to reason about the mental states of others (an ability called “Theory of Mind” or “ToM”). Since ToM was measured in this work by a test that requires participants to “read” the mental states of others from looking at their eyes (the “Reading the Mind in the Eyes” test), it is uncertain whether the same results would emerge in online groups where these visual cues are not available. Here we find that: (1) a collective intelligence factor characterizes group performance approximately as well for online groups as for face-to-face groups; and (2) surprisingly, the ToM measure is equally predictive of collective intelligence in both face-to-face and online groups, even though the online groups communicate only via text and never see each other at all. This provides strong evidence that ToM abilities are just as important to group performance in online environments with limited nonverbal cues as they are face-to-face. It also suggests that the Reading the Mind in the Eyes test measures a deeper, domain-independent aspect of social reasoning, not merely the ability to recognize facial expressions of mental states.

## Introduction

A growing body of research has been focused on the importance of individuals' ability to make inferences about others' mental states, termed “theory of mind” (ToM) or “mentalizing” [1]–[5]. A common assumption in much of this research is that people with greater ToM abilities will be more competent at various kinds of social interaction. But only a few studies have tested this in limited ways with children [6]–[8], and fewer still have tested it with adults [9]–[11].

One exception is a recent study by Woolley et al. [11], which found that groups of adults with higher average ToM scores also had significantly higher “collective intelligence,” as measured by the group’s ability to perform well across a wide range of different group tasks. Indeed, average ToM scores were the only significant predictor of collective intelligence in a regression that included other group composition and process variables.

However, it is unknown how rich interpersonal cues need to be in order for collective intelligence to emerge at all, or for individual members to engage in the mentalizing necessary to facilitate social interaction. Essentially all of the studies exploring the relationship between ToM and effective social interaction, including the Woolley et al. [11] study, have been conducted using face-to-face interactions. At the same time, an increasing proportion of social interactions today take place in online environments. According to the Pew Research Center, 73% of adults regularly use social networking sites of some kind, and 54% of American teenagers text their friends at least once a day, but only 33% talk to their friends face-to-face on a daily basis [12]. In addition to our social relationships, we increasingly work in online teams and rely on the collective intelligence of other groups whose collaboration is largely online [13], [14]. Online, many of the interpersonal cues that are important in face-to-face interactions (such as reading facial expressions and body language, or judging tone of voice) are not available, potentially impairing our ability to communicate effectively [15]–[17].

With this reduced communication bandwidth, it is to be expected that the ability to reason about the mental states of other group members should be impaired. This could reduce the predictive value of ToM for group performance in environments lacking in interpersonal cues; that is, ToM skills could be expected to play a much smaller role in online environments. On the other hand, one might also argue that ToM-related ability should be even more important in online interaction because the cues provided are so subtle that a lot of “reading between the lines” is necessary.

To help determine which of these views is correct, we compare the importance of ToM skills for predicting group performance in face-to-face and online groups. In so doing, we also test a new, web-based measure of collective intelligence that enables its measurement in both settings, allowing us to examine whether a collective intelligence emerges in online groups, and to replicate and extend previous findings [11].

## Collective Intelligence in Groups

Researchers have repeatedly demonstrated that a single statistical factor–often called “general intelligence” or “g”– emerges from the correlations among how well different people do a wide variety of different cognitive tasks (e.g., [18], [19]). This single factor can then be used to differentiate the characteristic performance levels of different individuals and to predict which individuals are likely to perform well on other tasks in the future.

In recent studies, Woolley et al [11] used the same statistical techniques used in individual intelligence research to see whether a similar collective intelligence factor exists for groups. In other words, they tried to determine the degree to which some groups are characteristically “smarter” than others across a wide range of tasks. To do this, they first gave different groups a variety of tasks that required qualitatively different collaboration processes [20], [21]. Then they used factor analysis to determine whether there was a single factor for a group–as there is for an individual–that predicts the group’s performance on all the different tasks.

They found that the first factor accounted for 43% of the variance in performance on all the different tasks. This is consistent with the 30–50% of variance typically explained by the first factor in a battery of individual cognitive tasks [18]. In individuals, this factor is called “intelligence” or “*g*.” For groups, Woolley et al. [11] called it “*collective intelligence*” or “*c*,” and it is a measure of the general effectiveness of a group on a wide range of tasks. The *c* factor was also shown to predict how well the groups performed more complex tasks at a later time, above and beyond the predictive ability of the average individual intelligence of group members.

They also found several factors that were significant predictors of *c*. First, the average and maximum intelligence of individual group members were correlated with *c,* but only moderately so. In other words, having a lot of smart people in a group did not necessarily make a smart group. Second, there was a significant correlation between *c* and the average ToM scores of group members, as measured by the “Reading the Mind in the Eyes” test [22]. Third, *c* was negatively correlated with the variance in the number of speaking turns by group members. In other words, groups where a few people dominated the conversation were less collectively intelligent than those with a more equal distribution of conversational turn taking. Finally, *c* was significantly correlated with the proportion of females in the group, with groups having more females being more collectively intelligent.

In addition, the researchers found that the effects of proportion of females were largely mediated by ToM scores since, consistent with previous research, women in the sample scored better on this measure than men. In a regression analysis including proportion of women, ToM scores, and conversational turn-taking, ToM scores remained the only significant predictor of collective intelligence.

Taken together, this research on collective intelligence suggests that the ToM abilities of group members play a large role facilitating group social interaction. What the existing research does not tell us is the degree to which these findings would apply in situations with highly impoverished communication channels such as many online environments. We do not even know, for instance, whether a single collective intelligence factor will emerge in online groups with limited communication, nor whether mentalizing ability will play as much of a role in that setting, due to the constraints on communication ability and the lack of interpersonal cues.

On the one hand, for example, previous research on collaboration in teams has found that the effect of using online tools for group tasks depends strongly on the type of tasks being performed [23]–[25]. If this effect dominates, we may see more variance in how the same group performs different tasks, and the factor analysis might result in several approximately equal factors for different types of tasks rather than a single dominant collective intelligence factor. On the other hand, if the most important determinants of group performance are the abilities of the members to work together, then we would expect a single important factor to emerge in online groups as was true with those working face-to-face.

## Theory of Mind and Emotional Intelligence

As mentioned previously, a large and growing body of research has focused on the importance of individuals' ability to make inferences about others' mental states, termed “theory of mind” (ToM), “mentalizing” [1]–[5], or, more recently, “mind reading” [26]. Such abilities are thought to be distinctly human, and fundamental to our ability to function in social settings. Indeed, severe impairment in ToM is a core characteristic of the developmental disorder of autism [2], [22].

Theory of mind is viewed by some as a subset of a broader array of skills and abilities associated with the more general concept of emotional intelligence (i.e., [27]). A growing body of work demonstrates the importance of emotional intelligence and related abilities to team performance [28]–[30]. Thus its connection with collective intelligence is consistent with existing work demonstrating the importance of the abilities of group members to recognize one another’s nonverbal emotional expression for group effectiveness [31], [32]. The broader construct of emotional intelligence not only encompasses social awareness (an ability closely associated with theory of mind) but also self-awareness, self-management, and relationship management [33]. Emotional intelligence has been empirically demonstrated to operate statistically as a second-stratum factor of cognitive ability, marking the expression of intelligence in the emotion domain [34]. However, little consensus exists regarding the best ways to measure the self-awareness and relationship management components of emotional intelligence, and when they are measured in reliable ways they tend to be highly correlated with one another [35], [36]. Theory of mind is, thus, among the small group of abilities within the broad category of emotional intelligence that can be most reliably measured. However, as discussed above, it remains unknown how much interpersonal information is needed for mentalizing to occur, or for such abilities to be relevant to facilitating social interaction. For instance, almost all previous studies of ToM and social interaction were conducted in settings permitting full face-to-face or verbal interaction [6]–[11]. Thus, the goal of this research is to explore the degree to which a collective intelligence factor arises even in temporary, online groups with highly constrained communication, and the degree to which ToM ability plays a role in facilitating collective intelligence in these settings.

## Method

A total of 272 participants were recruited from the general population via Internet advertisements in the Boston area and were invited to the laboratory and randomly assigned to a four-person team for a ninety-minute session. The Institutional Review Board at the Massachusetts Institute of Technology has reviewed and approved the study under COUHES protocol number 0801002578. The study was carried out in accordance with the declaration of Helsinki. All participants were paid for their participation and their written informed consent was obtained before the start of the session. Fifty-two percent of the participants were male.

We determined a priori that a minimum of 60 groups were needed to achieve at least 80% power to detect a medium to strong effect size, which we expected based on previous research [11]. During the time period that the facilities and participants were available to conduct the study, we were able to recruit enough participants for 68 groups. Each of the 68 groups was randomly assigned to one of two conditions: face-to-face or online. All participants were seated in front of personal laptop computers and worked with their group members in a shared online system to complete the tasks described below. Each face-to-face group was seated in a private room around a small table and was allowed to communicate freely. Members in the online groups were randomly seated in a large room with members of other groups. They did not know who else was in their group, and they only communicated with their group members via text chat in the shared online system. The entire group task battery was completed in just under an hour. Then all subjects completed individual tests, including the “Reading the Mind in the Eyes” (RME) test and individual personality scales.

### Measure of collective intelligence

**Figure 1:**
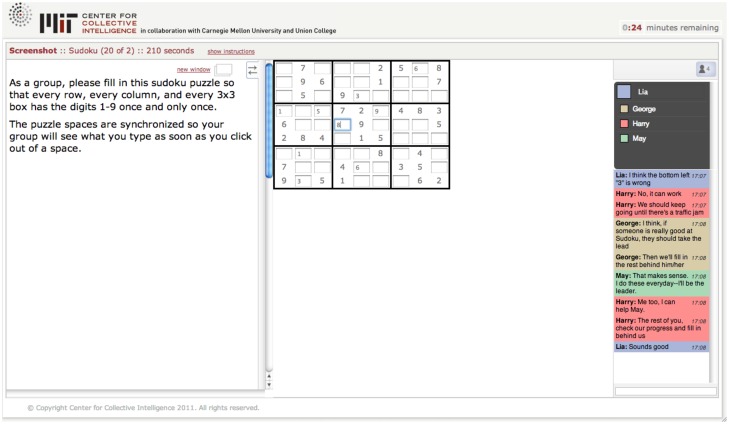
A screenshot of a group solving a Sudoku task. The right-hand chat window allows the group to communicate. The middle task panel is where the group enters its answers. The left-hand instructions panel contains instructions and other stimulus materials for the task.

Our measure of collective intelligence was an online (and significantly expanded) version of the approach used by Woolley et al [11]. The shared online system used to administer the test battery moved the group members through the tasks together, ensuring that all members of a group worked on the same tasks at the same time. Each task had a fixed duration of between 2 and 10 minutes, after which the next task would automatically begin. The entire battery took 64 minutes for a group to complete. Fig. 1 shows how a sample task in the test battery appeared on the screens of team members.

Our goal in task selection was to include a comprehensive sample of many different kinds of tasks that groups do. Accordingly, our initial selection was based on established taxonomies of team tasks [20], [21] and on the tasks used in prior studies of collective intelligence [11].

*Generating* tasks required the generation of new information and ideas. These included three brainstorming tasks (such as generating uses for a brick).

*Choosing* tasks required choosing among specified alternatives in two ways. In “intellective” tasks there was an objectively correct answer. These included an 18-item subset of the Raven’s Advanced Progressive Matrices test (a nonverbal test of abstract reasoning), a Sudoku puzzle task, a word unscrambling task, and a task of estimating quantities based on pictures. In “judgment” tasks, choosing among the alternatives was based on subjective judgments. These included predicting how a larger group of Americans would rate a series of slogans and images for quality on a ten-point scale.

*Executing* tasks required coordinated psychomotor performance across group members. These included typing tasks in which the group had to accurately transcribe into a shared online document as much as possible of a nonfiction text passage or a long list of numbers.

We also added two additional categories to reflect many other types of tasks important in the study of cognitive psychology, artificial intelligence, and groups (e.g., [37], [38], [39]).

*Remembering* tasks required groups to collectively store and retrieve information, similar to those evaluated in work on transactive memory systems [40], [41]. These included two memory tasks that tested memory for features from a video or a complex image.

*Sensing* tasks required groups to collectively detect patterns in an otherwise noisy environment. These included two detection tasks that presented grids of words or images in which the groups had to answer questions about the elements, e.g. finding the most frequent object.

We included at least one task from each category, as well as “verbal” and “nonverbal” variants of each category of tasks (see full descriptions of the tasks and additional descriptive statistics in the following Supplementary Materials available online: Task Descriptions; Tables S2, S3, S4a, and S4b in S1 Text).

Following Woolley et al. [11], we performed a factor analysis on all the groups’ scores on all the tasks. We call the first factor that emerges from this analysis the groups’ *collective intelligence.* Mathematically, scores on this collective intelligence factor represent weighted averages of the different task scores, with the weights derived from the correlations between the tasks and the first factor. Collective intelligence scores determined in this way have been shown to be strong predictors of the general effectiveness of a group on a range of more complex tasks [11].

### Group communication measures

We measured communication differently for face-to-face groups and online groups. For the face-to-face groups, we recorded each member’s spoken communication with individual microphones. Then we ran an automated script on these four audio tracks to determine who, if anyone, was speaking at each point in time. The total amount of communication was the total amount of time during which anyone was speaking. We also calculated the distribution of communication as the standard deviation in the amount of speaking time for different group members. For online groups, we calculated the total amount of communication as the total number of words in the online chat transcripts. We also calculated the distribution of communication as the standard deviation in the number of words chatted by different group members.

### Group member work contribution

Several of the tasks required groups to work together in a synchronized text editing environment (similar to that of Google Docs). For these tasks, we recorded how much text each member contributed to the final product and calculated the standard deviation of the members’ contributions.

### Reading the Mind in the Eyes (RME)

All participants completed the Reading the Mind in the Eyes test [22]. The test consists of 36 images of only the eyes of individuals, and participants are asked to choose among four possible mental states to describe the person whose eyes are pictured. The options are all complex mental states (e.g., shame, guilt, curiosity, desire) rather than simple emotions (e.g., happiness, anger). Our sample was similar in mean and variability on this test to the original general population sample in Baron-Cohen et al. [22] (ours: *M* = 24.85, *SD* = 5.24; original: *M* = 26.2, *SD* = 3.6). Individual scores of the group members were averaged for each group.

### Individual Personality Traits

Participants completed a Five Factor Personality Inventory [42], one of the most widely used personality tests. This test measures the five primary dimensions of adult personality: Extraversion, Agreeableness, Conscientiousness, Openness to Experience, and Neuroticism. Sixty items are responded to on a 1 to 5 scale; the mean for each scale was calculated for each participant. These scales were entered into a factor analysis to calculate a general factor of personality for each participant and aggregated to the group level, which was analyzed for its relationship with collective intelligence and RME [43].

## Results

**Figure 2:**
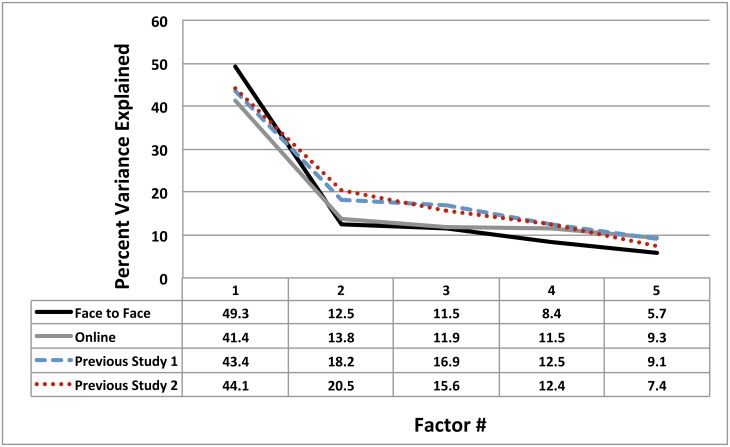
Scree plot comparing the variance explained by the first five factors extracted from a factor analysis of group performance on each of the groups tasks in this study, as compared to the factor structures reported in Woolley et al.'s [11] study. The first factor (collective intelligence) explains over 40% of the variance in all cases, while the second factor accounts for less than half of this amount, and the remaining factors account for much less.

First, replicating the results from Woolley et al. [11] using a new task battery and manipulating team member co-location, we found that the first factor in the factor analysis of group scores explained 49% of the variance in face-to-face and 41% in online groups, and the second factor explained less than half this amount (see Fig. 2). This provides additional support for the basic result from prior research that there is a single dominant collective intelligence factor for human groups, and that the existence of collective intelligence does not depend on groups being able to communicate face to face, but generalizes to online groups who communicate only by text. It also strengthens the evidence for collective intelligence by generalizing the finding from the test batteries used by Woolley et al. to the new battery developed here.

**Figure 3:**
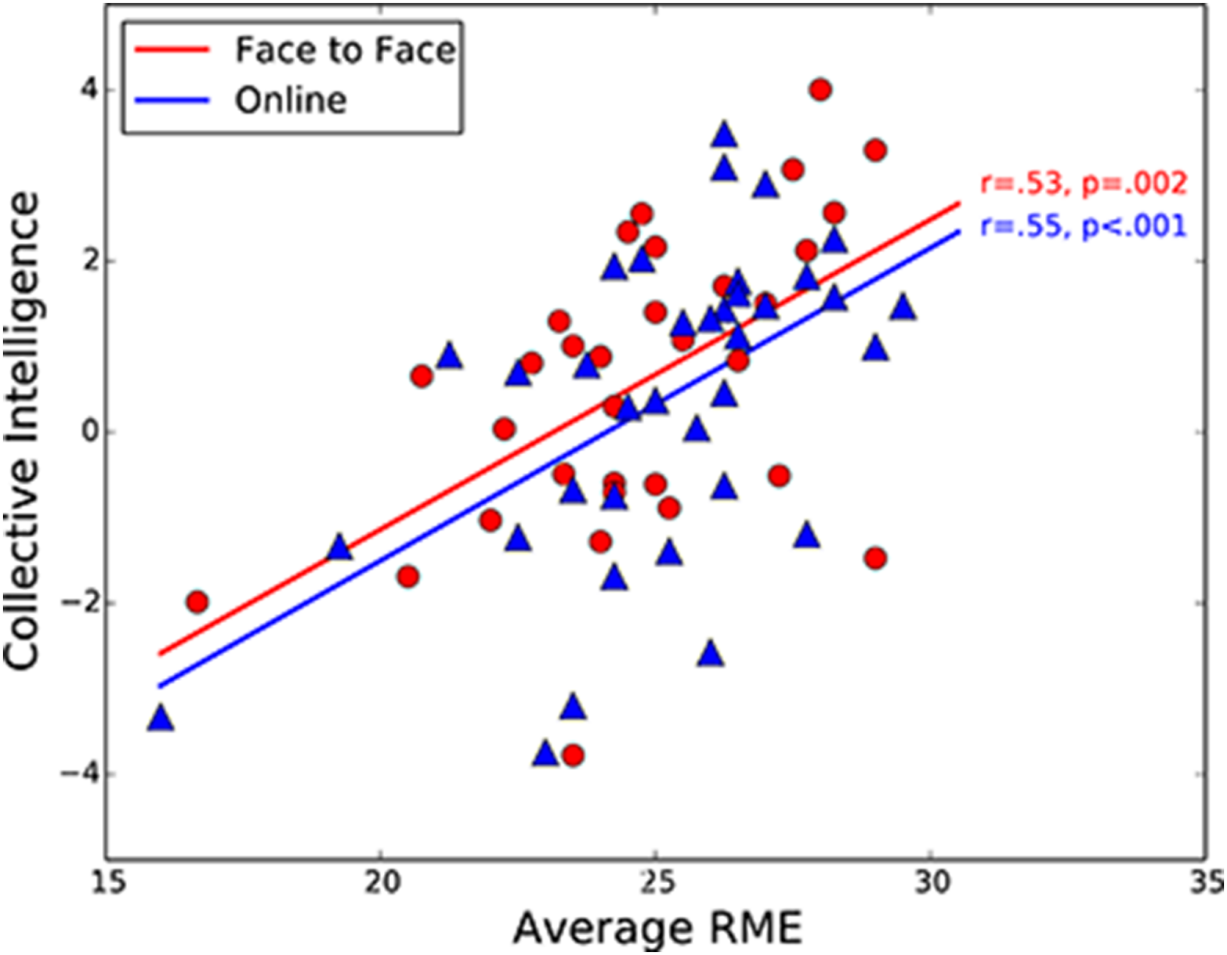
Relationship between average group RME and collective intelligence scores for face to-face (N = 32) and online (N = 36) groups. The correlations are significant and almost identical for both groups. Similar results are obtained when the two potential outlier groups with the lowest average RME scores are removed (r = .47, p<0.01 and r = .48, p<0.01 for face-to-face and online groups, respectively).

Surprisingly, we also found that not only did the average RME abilities of group members predict collective intelligence in the face-to-face condition (replicating the finding of Woolley et al. [11]), but they did so equally strongly in the online condition (Table 1; Fig. 3; *r* = .53, *p* = .002 for face-to-face groups; *r* = .55, *p*<.001 for online groups). In other words, the scores of group members on the RME measure were a strong predictor of how well the groups could perform a wide range of tasks together, even when participants were only collaborating online via text chat and could not see each other’s eyes or facial expressions at all. This suggests that scores on the RME test must be correlated with a broader set of ToM abilities and that these broader abilities are critical to group performance even when the subtle nonverbal cues available face-to-face (and operationalized in the RME test) are not available.

**Table 1:** Correlations between collective intelligence of a group and other group characteristics.

		CollectiveIntelligence	RME Test	%Female	Amount of Comm
**Online Groups**	*RME Test*	.55**			
	*% Female*	.41*	.36*		
	*Amount of Comm* 1	.47**	.33*	.10	
	*Distribution of* *Comm* 2	–.41*	–.45**	–.32*	–.25
**Face-to-Face Groups**	*RME Test*	.53**			
	*% Female*	.20	.16		
	*Amount of Comm* 1	.52**	.51**	–.06	
	*Distribution of Comm* 2	–.30*	–.25	.04	.01

* = p<.0.05.

** = p<0.01.

1 Amount of communication refers to chat communication in online groups and verbal communication in face-to-face groups.

2 Distribution of communication refers to the standard deviation of communication among members in a group.

These abilities also appear to be separate from traditional personality factors. We found no significant correlation between a general factor of personality [43] and collective intelligence or RME (*p*>.05 in both face-to-face and online conditions; see Table S4 in S1 Text).

In addition, we found that online groups were similar to face-to-face groups in other ways. First, we found that the proportion of women in the online groups was a strong predictor of collective intelligence (Table 1; *r* = .41, *p* = .01) and that the average RME ability in a group mediated the effects of the proportion of women in the group on collective intelligence (Sobel *z* = 1.95, *p* = .05), the same pattern we found in our previous study [11].

Next, we found that, in both face-to-face and online groups, the total amount of communication was positively correlated with collective intelligence, and the standard deviations of communication and work contribution were negatively correlated with collective intelligence (Table 1). In other words, groups that communicated more were more collectively intelligent, but groups in which one or two people dominated the discussion and activity were less collectively intelligent, whether the groups were online or face-to-face.

Finally, when we regressed collective intelligence onto the RME measure and the amount and distribution of communication within the group, we found these two factors have similar levels of influence on group performance in both the online and face-to-face conditions (see Table 2, models 1 and 4). This suggests that the theory of mind abilities associated with RME scores are facilitating group collaboration in ways beyond what is solely captured by the amount of verbal communication.

**Table 2:** Results of OLS Regressions of collective intelligence on the average Reading the Mind in the Eyes (RME) score, the amount of communication, and the distribution of communication.

	Condition 1: Face-to-Face			Condition 2: Online		
	1.	2.	3.	4.	5.	6.
Reading Mind in the Eyes	0.52**	0.34∧	0.26	0.55**	0.45**	0.38*
Amount of Comm		0.34∧	0.39∧		0.32*	0.30*
Distribution of Comm			−0.24			−0.17
F	8.44**	6.08**	4.81*	15.02**	10.91**	7.74*
R^2^	0.27**	0.36**	0.41**	0.31**	0.40*	0.42*
change R^2^		0.088∧	0.051		0.09**	0.02

∧ p<.10, two-tailed.

*p<.05, two-tailed.

**p<.001, two-tailed.

Amount and distribution of communication represent recorded verbal communication for face-to-face groups and text chat for online groups.

## Discussion

Taken together, these results provide strong empirical support for the emergence of collective intelligence in online groups, and the conclusion that theory of mind abilities are a significant determinant of group collective intelligence even when, as in many online groups, the group has extremely limited communication channels.

This study underscores the importance of continuing to develop our understanding of the factors contributing to collective intelligence in groups. Only recently have we come to understand collective intelligence as a unique quality of groups, indicative of their ability to perform a variety of different tasks [11]. Traditionally, a team’s potential has been conceptualized as the “resources” that are available to the group in the form of the information, intelligence, or other abilities of individual team members [44], [45] typically measured as the aggregate of individual members’ *g* or general intelligence [46]–[48] or the special expertise or task-specific cognitive abilities of team members [47], [49], [50].

The relationship between team cognitive ability and performance has been shown to vary with the way that cognitive ability is represented in the team and the type of task the team is performing. In particular, the performance of teams working on a task that requires a high degree of cooperation and communication is most influenced by the member with the *lowest* cognitive ability, because that person tends to slow the rest of the group [51]. In contrast, on tasks for which the optimal strategy is to select the best member (e.g., running a race, or answering a factual question), the cognitive ability of the highest scoring member predicts performance [44], [52]. Finally, more complex, multi-faceted tasks that require each member of the team to perform a subtask and then combine inputs into a team product are most influenced by the average ability of team members, because higher average cognitive ability is associated with greater propensity to adapt to a changing environment, as well as to learn from new information discovered in the course of work (e.g., [46], [53]).

However, it is also telling that the correlation of team average cognitive ability and performance drops to *r* = .14 in field settings, which is probably due to the fact that teams need to execute a range of functions that go beyond those that are predicted by aggregate cognitive ability [45], [47]. One probable explanation for the lack of explanatory power is the attempt to conceptualize *team* ability by aggregating *individual*-level measurements, which loses information in generalizing across levels of analysis [54]. This suggests that an index of a group's ability, such as the collective intelligence metric developed and tested here, utilizing uniquely group level information, should provide a better mechanism for predicting which groups will perform well in the future.

These results also demonstrate that teams comprised of members with a broader range of ability for perceiving subtle interpersonal cues will be better-equipped to develop higher levels of collective intelligence, especially in less rich, online chat-based environments. Here we used the RME test to measure theory of mind, but theory of mind ability can, in principle, be measured in many ways. The RME test is not the only measure of the construct; other tests include the Strange Stories test [55], the Faux Pas Recognition test [56], the Reading the Mind in the Voice test [57], and the Social Reasoning Wason Selection Test [58]. But the RME test is among the most widely accepted [59] and most well-validated in adult populations, having been shown to have adequate test-retest reliability [60] and to consistently differentiate control subjects from autism-spectrum individuals, who are below-average in social perceptiveness [22].

The RME test is also sensitive to differences between men and women and to differences among people who choose different educational fields [22]. The RME test has been shown to be sensitive in children to levels of prenatal testosterone [61] and in adults to administration of oxytocin [62]. All this suggests that the RME test is measuring a robust property of individual brain function; if performance on the test were completely determined by contextual factors then we would not expect to see such correlations or experimental effects. Crucially, unlike the other established ToM measures, which are designed for assessing children’s development, brain damage, or autism-spectrum conditions, the RME test has substantial variance in the general adult population, and it taps into both verbal and nonverbal aspects of interpretation in social situations.

The degree to which ToM, as measured by RME or otherwise, can be altered by training or experience remains an open question. Recent studies [63] suggest that theory of mind abilities as measured by RME can be, at least temporarily, improved by reading literary fiction, which implies a new and interesting avenue of research for improving group performance.

In summary, our results provide strong empirical support for the conclusion that even the collaboration of teams working online can be characterized by a single collective intelligence factor, and that theory of mind abilities are just as important to group effectiveness in these online environments where many kinds of non-verbal communication are not possible. In other words, it appears that the Reading the Mind in the Eyes test does not just measure the ability to read emotions in eyes but also the ability to “read between the lines” of text-based online interactions.

## Supporting Information

S1 Dataset **Dataset and glossary of the team-level scores for all tasks and metrics used in analyses.**
(ZIP)

S1 Text **Detailed descriptions of the tasks used in the collective intelligence battery.** Table S1, Task categories and verbal vs. non-verbal dimensions in the Collective Intelligence task battery. Table S2, Descriptive statistics of the task scores, group metrics, and the average scores for the individual metrics. Please refer to the text for a description of how the tasks are computed. Table S3, Correlations among the average Big 5 personality traits, the General Personality Factor [43], RME, the percent of Females in the group (% Female), and collective intelligence (CI). Table S4a, Correlations among collective intelligence (CI), task scores, RME, and interaction metrics for face-to-face groups. Table S4b, Correlations between collective intelligence (CI), task scores, RME, and interaction metrics for online groups.
(DOCX)
