# Supplementary material for: Reading the Mind in the Eyes or Reading between the Lines? Theory of Mind Predicts Collective Intelligence Equally Well Online and Face-To-Face
Source: PLoS One. 2014 Dec 16;9(12):e115212. doi: 10.1371/journal.pone.0115212 (PMC4267836; doi:10.1371/journal.pone.0115212)
Supplement: S1 Text — Detailed descriptions of the tasks used in the collective intelligence battery. Table S1, Task categories and verbal vs. non-verbal dimensions in the Collective Intelligence task battery. Table S2, Descriptive statistics of the task scores, group metrics, and the average scores for the individual metrics. Please refer to the text for a description of how the tasks are computed. Table S3, Correlations among the average Big 5 personality traits, the General Personality Factor [43], RME, the percent of Females in the group (% Female), and collective intelligence (CI). Table S4a, Correlations among collective intelligence (CI), task scores, RME, and interaction metrics for face-to-face groups. Table S4b, Correlations between collective intelligence (CI), task scores, RME, and interaction metrics for online groups. (DOCX) [file pone.0115212.s002.docx]

**Task Descriptions**

**Group tasks:** *Brainstorming (Category 1).* Groups had to complete three brainstorming tasks. In the first one, the task required that the group come up with as many words as possible that start with S and end in N. The second task asked groups to brainstorm different uses of a brick. The final one asked groups to brainstorm as many equations as possible that equal 10 while using the operators +,–,/,* and using the numbers between 2 and 8 only once per equation. For all brainstorming tasks, one point was awarded for each correct answer plus up to an additional point if the answer was rare across all the groups. Repeated answers within a group were penalized through loss of points. The groups were informed about these scoring rules before the task. The groups had three minutes to complete each of the three brainstorming tasks.

*Unscramble Words (Category 2).* Groups had two minutes to unscramble as many words as possible on a list of 24 randomly scrambled words. Each item on the list had only one correct answer, and groups received one point for each correct answer.

*Group Matrix Reasoning (Category 2).* Raven’s Advanced Progressive Matrices (RAPM) is a standardized test of general fluid reasoning capacity. Participants were given five minutes to complete half of the 36-item Raven’s II test (all of the odd-numbered items). Each item presents a 3 x 3 array (or “matrix”) of items with the lower-right corner empty. Based on patterns in the array, subjects must pick which of eight answer pieces properly belongs in the empty space. There is only one correct answer per question. Questions become progressively more difficult as the test goes on. Examples of items are available from Harcourt Education.

*Sudoku (Category 2).* Sudoku is a popular logical reasoning game where a 9x9 grid (subdivided into nine 3x3 boxes) is partially filled with numbers from 1 to 9. Each number can only appear once per column, once per row, and once in each 3x3 box. A proper Sudoku grid has one unique solution and one correct number for each grid space. Groups were given five minutes to cooperatively solve a Sudoku on our synchronized interface. One point was awarded for each correct grid space.

*Judgment (Category 2).* For the first two judgment tasks, the groups were asked to predict how a larger population would judge a stimulus. The first stimulus was a series of products and accompanying slogans. The group had to rate these slogans on a scale of 1 (worst) to 10 (best) based on what they believed a large sample of Americans would rate their suitability for the given products. Altogether, there were 4 products with 3 slogans each, resulting in 12 total slogans for groups to rate. The ground truth was based on poll responses of 100 American users on Amazon Mechanical Turk. The groups received points based on how close their ratings were to the ground truth averages. The second judgment task involved rating 5 pictures on a scale of 1 (worst) to 10 (best) based on what participants believed a large sample of Americans rated them. The ground truth and the scoring of this task were established in the same way as with the slogan judgment task. For the third judgment task, groups were asked to judge the number of pages in books based on images of their page edges. The groups received points proportional to the number of book pages that their estimate erred, corrected for the total number of pages in the book (i.e. 10 pages wrong out of a 400-page book is a smaller error than 10 pages wrong out of a 50-page book). The combined time for all judgment tasks was 10 minutes.

*Group Typing (Category 3).* Groups were given either a complex text or a complex field of numbers and worked together to type as much of it as possible into a shared online document, similar to a Google Doc, in seven minutes. Teams earned one point for each word/number correctly typed and lost one point for each word/number skipped or for each typo. As a result, team members needed to carefully coordinate their work so as not to type over the work of other members, skip whole sections, or mix up the order of paragraphs and sentences. These errors all resulted in the loss of many points.

*Memory (Category 4).* The battery included three memory tasks. The first one was a video memory task in which group members were shown a 90-second video three times and then answered a set of questions about its features afterwards. They received points for each correct answer. The second task was exactly the same as the first one, except that group members were shown a complex image instead and answered questioned about its features. The third task was a memory words task where groups needed to remember a set of 50 words. However, there were substantial differences in the interpretation of the rules for this task among the groups, and thus we excluded this task before evaluation. The total time for all memory tasks was 11 minutes.

*Detection (Category 5).* In a series of detection tasks, the groups were presented either with a large grid of words or with a large grid of images. They were asked to judge the characteristics of the grid: “What is the most frequent object in the grid?”, “Which is the least frequent object in the grid?”, and “Which two objects have the most similar frequency?”. The time limit was 90 seconds per grid in order to make it impossible for any individual member to exhaustively count all objects in the grid. Groups received points for the correct answer and partial credit based on how close their answers were to the correct answers.

| **Table S1** |  | |  |
| --- | --- | --- | --- |
| Task categories and verbal vs. non-verbal dimensions in the Collective Intelligence task battery. | | | |
| **Task Category** | | **Verbal** | **Non-Verbal** |
| 1. Generating | | Brainstorming Words | Brainstorming Uses for a Brick Brainstorming Equations |
| 2. Choosing | | Unscramble Words  Judgment Slogans | Matrix Reasoning  Sudoku  Judgment Picture  Judgment Pages |
| 3. Executing | | Typing Text | Typing Numbers |
| 4. Remembering | | Memory Words^1^ | Memory Video  Memory Images |
| 5. Sensing | | Detection Words | Detection Images |

^1^ Due to technical problems with administration of this task it was excluded from the analysis.

| **Table S2** |  |  |  |  |
| --- | --- | --- | --- | --- |
| Descriptive statistics of the task scores, group metrics, and the average scores for the individual metrics. Please refer to the text for a description of how the tasks are computed. | | | | |
|  | **Minimum** | **Max** | **Mean** | **SD** |
| Collective Intelligence (c) | -2.41 | 2.08 | 0 | 1 |
| Brainstorming Words | 1.39 | 83.92 | 22.28 | 12.82 |
| Brainstorming Uses for a Brick | 2.23 | 49.65 | 18.45 | 9.39 |
| Brainstorming Equations | 0.00 | 21.00 | 7.77 | 4.53 |
| Unscramble | 0 | 10 | 4.92 | 2.4 |
| Matrix Reasoning | 0 | 10 | 4.91 | 2.17 |
| Sudoku | 0 | 42 | 16.56 | 10.65 |
| Judgment Slogans | -3.40 | 20.40 | 11.17 | 4.49 |
| Judgment Pictures | -9.49 | 17.61 | 10.42 | 4.85 |
| Judgment Book Pages | 48 | 352 | 221.39 | 64.53 |
| Detection Images | 0 | 10 | 3.95 | 2.31 |
| Detection Words | 0 | 7 | 2.48 | 1.96 |
| Memory Video | 0 | 5 | 2.33 | 1.36 |
| Memory Image | 0 | 5 | 2.57 | 1.27 |
| Typing Text | 12.03 | 781.43 | 245.71 | 161.38 |
| Typing Numbers | 21.08 | 868.00 | 309.27 | 163.37 |
| Reading Mind in the Eyes | 15 | 33 | 24.93 | 3.58 |
| Portion Female | 0 | 1 | 0.45 | 0.33 |
| SD Text Pad Edits | 0.01 | 0.18 | 0.063 | 0.04 |
| Amount of communication (1) | 6576 | 13143 | 9113.42 | 1652.65 |
| Distribution of communication (2) | 0.02 | 0.14 | 0.07 | 0.03 |
| Amount of communication Count (3) | 13 | 462 | 226.97 | 106.2 |
| Distributionn of communication (4) | 0.04 | 0.22 | 0.11 | 0.04 |

- 1. Amount of verbal communication in face-to-face groups
  2. Standard deviation among individuals of verbal communication in face-to-face groups
  3. Amount of text chat communication in online groups
  4. Standard deviation among individuals of text chat communication in online groups

| **Table S3** |  |  |  |  |  |  |  |  |  |
| --- | --- | --- | --- | --- | --- | --- | --- | --- | --- |
| Correlations among the average Big 5 personality traits, the General Personality Factor (Woodley & Bell, 2011), RME, the percent of Females in the group (% Female), and collective intelligence (CI). | | | | | | | | | |
|  | **CI** | **1** | **2** | **3** | **4** | **5** | **6** | **7** | **8** |
| (1) Extraversion | -.09 |  |  |  |  |  |  |  |  |
| (2) Agreeableness | .26* | .47** |  |  |  |  |  |  |  |
| (3) Conscientiousness | -.14 | .22 | .24 |  |  |  |  |  |  |
| (4) Stability | -.15 | .26* | .40** | .28* |  |  |  |  |  |
| (5) Openness | -.01 | .30* | .15 | .11 | .18 |  |  |  |  |
| (6) General Personality Factor^1^ | -.21 | .73** | .75** | .54** | .68** | .48** |  |  |  |
| (7) RME | .53** | .17 | -.05 | .01 | -.09 | .29* | .08 |  |  |
| (8) % Female | .31* | .03 | .16 | .05 | -.07 | .05 | .07 | .25* |  |
| (9) Team Cohesion | .09 | .11 | .23 | -.05 | .10 | .09 | .16 | .06 | .32** |
| ^1^The factor analysis of the Big 5 personality variables yielded a single factor with an eigenvalue > 1.0, (eigenvalue = 2.08) and the correlations of each of the individual personality variables are equivalent to the factor loadings on this single factor.  * Correlation is significant at p<.05, two-tailed ** Correlation is significant at p<.01, two-tailed | | | | | | | | | |

| **Table S4a** |  |  |  |  |  |  |  |  |  |  |  |  |  | |
| --- | --- | --- | --- | --- | --- | --- | --- | --- | --- | --- | --- | --- | --- | --- |
| Correlations among CI, task scores, RME, and interaction metrics for face-to-face groups | | | | | | | | | | | | | |  |
|  | **1** | **2** | **3** | **4** | **5** | **6** | **7** | **8** | **9** | **10** | **11** | **12** | **13** | |
| (1) Collective Intelligence (c) |  |  |  |  |  |  |  |  |  |  |  |  |  | |
| (2) Brainstorm | 0.78** |  |  |  |  |  |  |  |  |  |  |  |  | |
| (3) Unscramble | 0.62** | 0.49** |  |  |  |  |  |  |  |  |  |  |  | |
| (4) Matrix Solving | 0.78** | 0.61** | 0.54** |  |  |  |  |  |  |  |  |  |  | |
| (5) Sudoku | 0.80** | 0.64** | 0.45** | 0.6** |  |  |  |  |  |  |  |  |  | |
| (6) Judgment | 0.68** | 0.51** | 0.25 | 0.47** | 0.45** |  |  |  |  |  |  |  |  | |
| (7) Detection | 0.52** | 0.38* | 0.18 | 0.48** | 0.20 | 0.42* |  |  |  |  |  |  |  | |
| (8) Memory | 0.47** | 0.16 | 0.16 | 0.18 | 0.23 | 0.26 | 0.16 |  |  |  |  |  |  | |
| (9) Typing | 0.84** | 0.54** | 0.4* | 0.57** | 0.64** | 0.56** | 0.36* | 0.41* |  |  |  |  |  | |
| (10) RME | 0.53** | 0.52** | 0.22 | 0.54** | 0.53** | 0.54** | 0.20 | -0.04 | 0.41* |  |  |  |  | |
| (11) % Female | 0.20 | 0.08 | 0 | 0.18 | 0.36* | -0.21 | -0.09 | 0.22 | 0.31 | 0.16 |  |  |  | |
| (12) Amount Speaking | 0.52** | 0.52** | 0.24 | 0.45* | 0.44* | 0.41* | -0.11 | 0.23 | 0.44* | 0.51** | -0.06 |  |  | |
| (13) SD Speaking | -0.30 | -0.21 | -0.25 | -0.33 | -0.18 | -0.19 | -0.15 | 0.11 | -0.36 | -0.25 | 0.04 | 0.01 |  | |
| (14) SD Edits | -0.47** | -0.37* | -0.13 | -0.33 | -0.4* | -0.22 | -0.03 | -0.51** | -0.47** | -0.37* | -0.42* | -0.38 | 0 | |
| * Correlation is significant at p<.05, two-tailed ** Correlation is significant at p<.01, two-tailed  SD Speaking and SD Edits are the standard deviation of communication and standard deviation of contributions to the tasks, respectively | | | | | | | | | | | | |  | |

| **Table S4b** |  |  |  |  |  |  |  |  |  |  |  |  |  |
| --- | --- | --- | --- | --- | --- | --- | --- | --- | --- | --- | --- | --- | --- |
| Correlations between CI, task scores, RME, and interaction metrics for online groups | | | | | | | | | | | | | |
|  | **1** | **2** | **3** | **4** | **5** | **6** | **7** | **8** | **9** | **10** | **11** | **12** | **13** |
| (1) Collective Intelligence (c) |  |  |  |  |  |  |  |  |  |  |  |  |  |
| (2) Brainstorm | 0.75** |  |  |  |  |  |  |  |  |  |  |  |  |
| (3) Unscramble | 0.61** | 0.53** |  |  |  |  |  |  |  |  |  |  |  |
| (4) Matrix Solving | 0.74** | 0.52** | 0.35* |  |  |  |  |  |  |  |  |  |  |
| (5) Sudoku | 0.63** | 0.26 | 0.37* | 0.23 |  |  |  |  |  |  |  |  |  |
| (6) Judgment | 0.41* | 0.24 | 0.13 | 0.24 | 0.17 |  |  |  |  |  |  |  |  |
| (7) Detection | 0.48** | 0.31 | 0.29 | 0.19 | 0.34* | 0.03 |  |  |  |  |  |  |  |
| (8) Memory | 0.75** | 0.34* | 0.27 | 0.56** | 0.6** | 0.23 | 0.26 |  |  |  |  |  |  |
| (9) Typing | 0.67** | 0.51** | 0.12 | 0.58** | 0.20 | 0.19 | 0.30 | 0.47** |  |  |  |  |  |
| (10) RME | 0.55** | 0.44** | 0.28 | 0.31 | 0.43** | 0.19 | 0.34* | 0.4* | 0.41* |  |  |  |  |
| (11) % Female | 0.41* | 0.24 | -0.12 | 0.33 | 0.41* | 0.40* | 0.24 | 0.34* | 0.31 | 0.36* |  |  |  |
| (12) Chat Count | 0.42** | 0.41* | 0.29 | 0.41* | 0.06 | 0.19 | 0 | 0.30 | 0.42* | 0.31 | 0.01 |  |  |
| (13) Distribution of Chat | -0.41* | -0.45** | -0.15 | -0.29 | -0.33* | -0.16 | -0.13 | -0.27 | -0.25 | -0.45** | -0.32 | -0.16 |  |
| (14) SD Edits | -0.42* | -0.34* | -0.20 | -0.26 | -0.31 | -0.09 | -0.34* | -0.26 | -0.36* | -0.35* | -0.31 | -0.29 | 0.45** |
| * Correlation is significant at p<.05, two-tailed ** Correlation is significant at p<.01, two-tailed  Distribution of Chat is the Standard Deviation of the lines of chat  SD Edits is the standard deviation of contributions to the tasks among members | | | | | | | | | | | | | |
